# Evidence of Olfactory Imprinting at an Early Life Stage in Pink Salmon (*Oncorhynchus gorbuscha*)

**DOI:** 10.1038/srep36393

**Published:** 2016-11-09

**Authors:** Nolan N. Bett, Scott G. Hinch, Andrew H. Dittman, Sang-Seon Yun

**Affiliations:** 1 University of British Columbia, Department of Forest and Conservation Sciences, 2424 Main Mall, Vancouver, BC, V6T 1Z4, Canada; 2 Environmental and Fisheries Sciences Division, Northwest Fisheries Science Center, National Marine Fisheries Service, NOAA, 2725 Montlake Boulevard East, Seattle, WA, 98112, USA; 3 University of British Columbia, Faculty of Land and Food Systems, 2357 Main Mall, Vancouver, BC, V6T 1Z4, Canada

## Abstract

Pacific salmon (*Oncorhynchus* spp.) navigate towards spawning grounds using olfactory cues they imprinted on as juveniles. The timing at which imprinting occurs has been studied extensively, and there is strong evidence that salmon imprint on their natal water during the parr-smolt transformation (PST). Researchers have noted, however, that the life histories of some species of Pacific salmon could necessitate imprinting prior to the PST. Juvenile pink salmon (*O. gorbuscha*) spend less time in fresh water than any other species of Pacific salmon, and presumably must imprint on their natal water at a very young age. The time at which imprinting occurs in this species, however, has not been experimentally tested. We exposed juvenile pink salmon as alevins to phenethyl alcohol (PEA) or control water, reared these fish to adulthood, and then tested their behavioural responses to PEA to determine whether the fish successfully imprinted. We found that pink salmon exposed to PEA as alevins were attracted to the chemical as adults, suggesting that imprinting can occur during this stage. Our finding provides some of the first evidence to support the long-standing belief that imprinting can occur in pink salmon prior to the PST.

The ability of Pacific salmon (*Oncorhynchus* spp.) to home to their natal stream is an iconic example of animal navigation during a large scale migration. Wisby and Hasler^1^ hypothesized that juvenile salmon ‘imprint’ on the unique odour of their natal water, and returning adults use their sense of smell to detect these imprinted odours. Subsequent research confirmed the occurrence of olfactory imprinting^2^,^3^, and suggested that the memory of the natal stream is formed during the parr-smolt transformation (PST), as juvenile salmon prepare for their downstream migration to the ocean^4^,^5^,^6^,^7^,^8^. Most of the research on the timing of imprinting has focused on coho salmon (*O. kisutch*) but imprinting during the PST has been confirmed in other species as well, including steelhead (*O. mykiss*)^9^, Atlantic salmon (*Salmo salar*)^10^ and brown trout (*S. trutta*)^11^. Concurrent with the imprinting activity during the PST is a surge in circulating thyroxine^12^ that may regulate the imprinting process^13^,^14^. A recent study^15^ on chum salmon (*O. keta*) also found increased expression of the NR1 subunit of the N-methyl-D-aspartate receptor, which influences memory formation in fish and could be critical to successful imprinting, during the downstream migration.

Although imprinting occurs during the PST in species that rear for a period of time in their natal streams, such as coho salmon, species with different life histories must imprint earlier during development^6^,^16^,^17^. Pink salmon (*O. gorbuscha*) and chum salmon, for example, leave their natal area and swim towards the ocean shortly after emergence from the incubation site, while sockeye salmon (*O. nerka*) swim directly to a lake. For these species, homing to the natal stream presumably requires learning home stream odours prior to leaving the incubation site, perhaps before emergence or even hatching. Indeed, there is evidence that sockeye salmon fry learn the odour of their incubation site before leaving for a lake^18^,^19^, and that some form of learning occurs in sockeye salmon as early as the embryo stage^20^. The most direct evidence of imprinting during an early life stage was provided by Tilson *et al.*^21^, who demonstrated imprinting in sockeye salmon during the alevin stage (when the developing salmon has hatched, but retains nutrients from the egg in a yolk sac and remains in the gravel of the nest site) and the emergence stage (when the yolk sac has been fully absorbed, and the salmon emerges from the gravel). They exposed individuals to a synthetic chemical during several periods of their early development, and found increased whole body thyroxine levels during the alevin and emergence stages, as well an attraction to the imprinting chemical in adults exposed during those periods.

Unlike sockeye salmon, however, which typically reside one or two years in a lake that may share some of the odours from the home stream, pink and chum salmon are the only species of Pacific salmon that do not rear in fresh water before migrating to the ocean. Pink salmon in particular leave their natal sites quickly, developing salinity tolerance around the time of yolk-sac absorption^22^, and spending less time in fresh water after emerging from the gravel than any other *Oncorhynchus* species^23^. Natal site fidelity appears to be lower in pink salmon than the other species as well^24^, which could result from an imprinting period that is contracted or occurs at an earlier developmental stage, as necessitated by their unique life history. Aside from the research of Tilson *et al.*^21^, though, direct evidence of imprinting during larval stages is limited^25^, and olfactory imprinting prior to emergence from the gravel has not been demonstrated in pink salmon.

We attempted to imprint juvenile pink salmon to the odour of phenethyl alcohol (PEA), a chemical that has been used extensively in previous imprinting studies. These studies demonstrated that juvenile salmon can learn specific chemicals such as PEA and later use these chemicals to guide homing adults^2^,^3^,^6^,^26^. Furthermore, PEA is a natural compound^27^,^28^ that elicits olfactory responses^7^,^26^,^29^ and can be learned by salmon^6^,^26^ and other fish species^30^,^31^. Unlike many odorants such as amino acids, PEA is also ideal for imprinting studies because it does not elicit innate behavioural responses^2^,^31^ and is not typically present in municipal waters (used as control water in these studies) allowing for precise control of the concentration. We reared offspring from wild Fraser River pink salmon in a laboratory, and exposed them to either control water or water containing PEA during the alevin, stage. We then tested the behavioural response of these fish to PEA as two-year-old adults during the time of their natural spawning migration. We predicted that fish would imprint to PEA during the alevin stage, the period that directly precedes their emigration from their natal site.

## Methods

### Experimental Animals

As part of an unrelated study examining swim performance in juvenile pink salmon, a small number of wild pink salmon adults were sampled for sperm and eggs from fish that arrived at artificial spawning grounds in Seton River and Weaver Creek, both tributaries of the lower Fraser River, B.C., Canada, in October 2013. They are situated 215 river km from each other and spawning occurs at the same times of the year in both channels. The population structure of pink salmon in the lower Fraser River is not well defined (as it is with other species such as sockeye salmon) and individuals spawn throughout this entire region at the same time. The gametes were transported to a laboratory at the University of British Columbia where the eggs were fertilized (15 females were each crossed with 3 males for each of the two populations, and a random subset from these were used for this experiment), incubated at 8 °C in Heath trays until emergence (i.e. full yolk sac absorption), and then transferred to 1000 l flow-through troughs, separated by population and treatment. In December 2014, the fry were implanted with Passive Integrated Transponder (PIT) tags (that emit a unique identification code), and in April 2015 they were transferred to a communal 8000 l flow-through tank. They remained in the tank until behavioural trials. Throughout the experiment, lighting was adjusted to match the photoperiod of their natural environment, and water temperatures fluctuated naturally over the course of the two years from 6 °C to 18 °C. The fish were fed commercial salmon feed (EWOS Canada Ltd., BC, Canada) ad libitum until behavioural trials.

### Odour exposures

Fish were reared and tested in dechlorinated municipal water originating from Metro Vancouver’s Capilano watershed. During the alevin stage (December 15, 2013 to January 28, 2014; 603–859 accumulated temperature units), fish were continuously exposed to control water or water containing 10^−7 ^M PEA (W285803 Sigma-Aldrich). A stock solution of 10^−5 ^M PEA dissolved in municipal water was metered into the Heath trays using a peristaltic pump (Masterflex L/S model 7536–04, Cole-Parmer, QC, Canada) to achieve a final concentration 10^−7 ^M PEA. This concentration of PEA has been used in previous salmon imprinting studies^3^,^6^.

### Behavioural Trials

**Figure 1:**
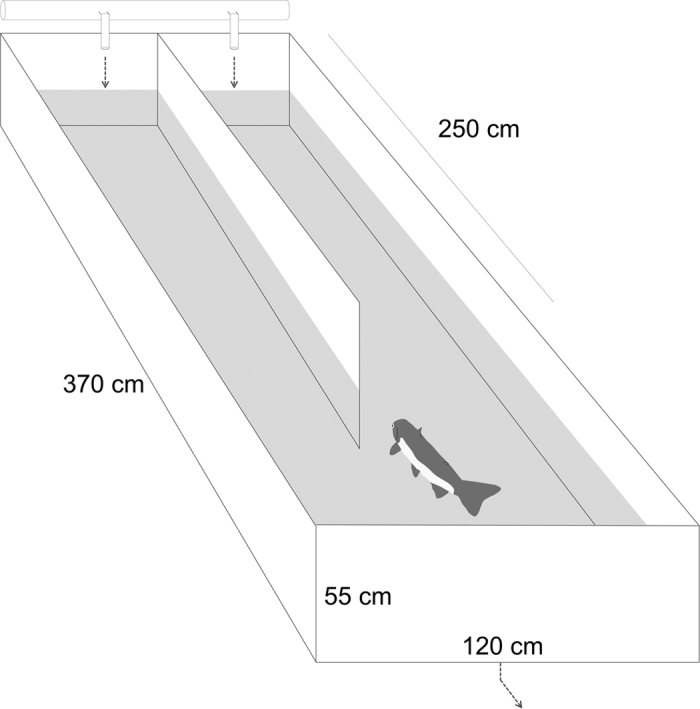
The two-choice chamber, or Y-maze, used to conduct behavioural tests on adult pink salmon. The imprinting chemical, PEA, was added to one of the arms and the behaviour of the salmon was recorded using an underwater video camera.

We conducted behavioural trials from October 15 to October 27, 2015. We used a two-choice chamber, or Y-maze, to test the behavioural responses of the adult pink salmon to PEA (Fig. 1). The Y-maze was constructed from plywood and sealed with fish-safe liquid rubber (Ames Research Laboratories, Salem, OR), and was covered with a tarp. Water entered each side, or arm, of the Y-maze at a rate of 15 L min^−1^, and a dye test demonstrated that no water was exchanged between the two arms. Using the peristaltic pump, a stock solution of 10^−4 ^M PEA was added from a 20 L acid-washed Nalgene container to one arm of the Y-maze to achieve a final concentration of 10^−7 ^M. The solution was dripped into the inlet water as it flowed into the maze. Water from a second 20 L acid-washed Nalgene container was added to the other arm at the same rate. The water was drained and refilled between trials, and the arm receiving PEA was alternated daily such that the number of trials with PEA in each arm was equivalent.

At the start of each trial, a pink salmon was transferred from the rearing tank to the downstream end of the Y-maze. A plastic gate prevented the fish from entering the two arms. After a 20 minute acclimation period, the gate was removed and the fish’s activity was recorded for 30 minutes using an underwater camera (Cameray Electronic Co., Ltd., China). Behaviours were monitored visually, rather than through the use of a PIT array as in some studies e.g. ref. 32, to capture behaviours that would be missed by an automated PIT system. We recorded the amount of time each fish spent in each of the two arms, as well as the number of times each fish crossed through the stream of water flowing into each arm. The latter behaviour captured the activity of the fish at the upstream end of the arm and could indicate an attempt to swim further ‘upstream’, as salmon are positively rheotactic during the spawning migration^33^. After the trial, we euthanized each fish with tricaine methanesulfonate (MS-222), measured the fish’s fork length and weight, and dissected each fish to confirm sex. The Y-maze was drained and refilled between trials to limit the possibility of chemical cues released by the fish influencing subsequent trials.

The fork lengths and weights (mean ± standard error) of the salmon at the time of trials were 29.3 ± 0.7 cm and 270 ± 17 g for females (*n* = 16), and 28.1 ± 0.5 cm and 267 ± 16 g for males (*n* = 12). We ran the tests when the salmon were clearly nearing maturity and developing secondary sex characteristics. The time of year was also consistent with when these fish would be migrating and spawning in the Fraser River.

The research conformed to protocols approved by the University of British Columbia Committee on Animal Care (A15-0205) and met the Canadian Council for Animal Care guidelines.

### Statistical Analysis

Due to mechanical issues with our rearing facility, we were not able to rear large numbers of fish to adulthood. To ensure adequate sample sizes for analyses, we pooled fish whose parents came from the two adult spawning channel sources. This should not bias the core results as the two parent sources are nearly identical in their freshwater imprinting timing and duration. Based on acoustic tagging results, juvenile sockeye salmon migrate down the Fraser River at an average speed of 150 km per day^34^, which is the approximate speed of the Fraser River in spring. Assuming pink salmon also travel at the speed of the river, juvenile Seton River pink salmon, which are spawned further up the Fraser River than juvenile Weaver Creek pink salmon, would only take ~1.5 days longer to migrate to the ocean than Weaver Creek juveniles. This, combined with the fact that juveniles from both channels immediately migrate downstream following emergence, and do so at the same time of year, suggests that their imprinting patterns are similar. We compared the amount of time spent in each arm using paired t-tests (α = 0.05), and the proportion of time spent in the arm with PEA using a one sample t-test (μ = 0.5, α = 0.05), both of which met assumptions of variance and normality. We compared the number of times the fish crossed through the inflow of the water in each arm, which did not meet assumptions of normality even following transformation, with a Wilcoxon signed-rank test (α = 0.05). Statistical analyses were performed in R Studio (V 0.98.501, RStudio Inc., Boston, MA).

## Results

**Figure 2:**
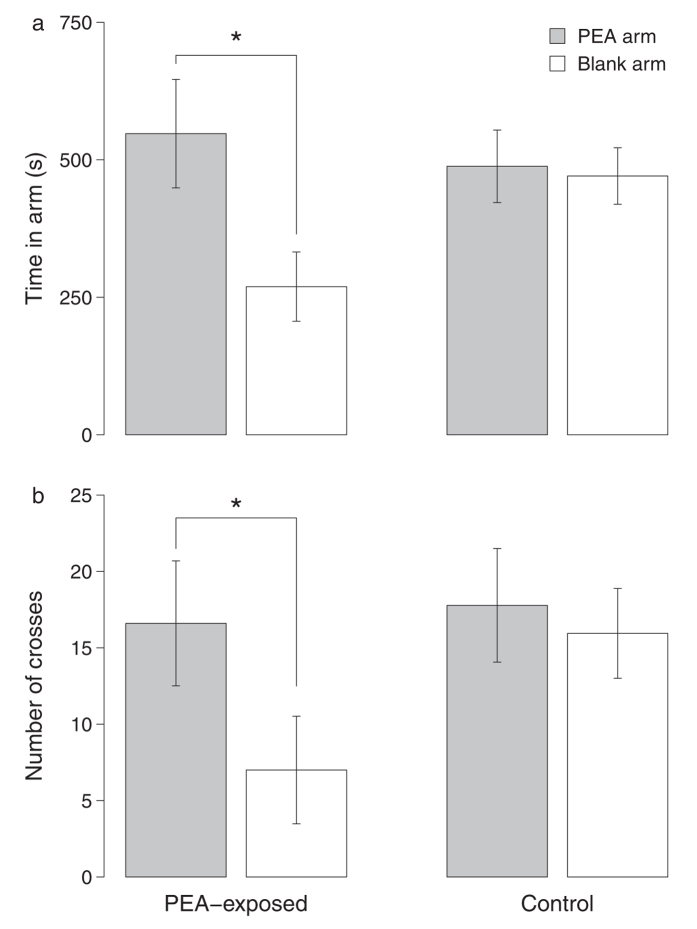
Behavioral responses of pink salmon to PEA, an imprinted chemical. Responses include (**a**) the amount of time adult pink salmon spent in the arm containing PEA and the arm lacking PEA and (**b**) the number of times the fish crossed through the stream of water entering into each arm. Fish in the “PEA-exposed” group were exposed to PEA during the alevin stage, while fish in the “control” group were never exposed to PEA. Adult salmon are positively rheotactic, and movement through the water flowing into the Y-maze may indicate an attempt to swim further into the current in this direction. Asterisks indicate significant differences (*P* < 0.05).

**Figure 3:**
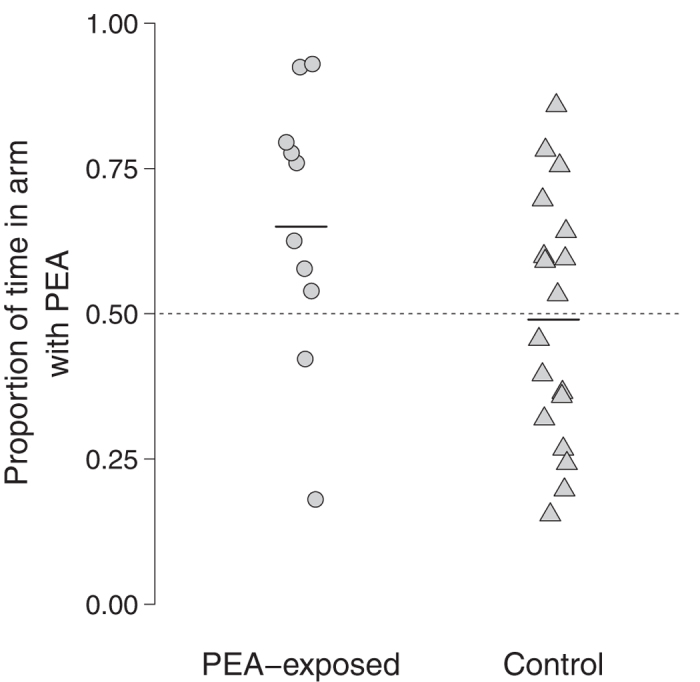
The proportion of time spent by adult pink salmon in the arm containing PEA. Fish in the “PEA-exposed” group were exposed to PEA during the alevin stage, while fish in the “control” group were never exposed to PEA. The dotted horizontal line represents a value of 0.5, i.e. an equal proportion of time spent in this arm as in the arm lacking PEA. The solid horizontal lines represent the mean values.

Adult pink salmon exposed to PEA during the alevin stage (*n* = 10; 6 females and 4 males) spent more time in the arm containing PEA (*t*_9_ = 2.27, *P* = 0.049; Fig. 2a), and they also crossed through the stream of water flowing into this arm more frequently than the stream flowing into the other arm (*V* = 40, *n* = 10, *P* = 0.044; Fig. 2b). The proportion of time spent in the arm with PEA was also greater than 0.5 in this group, although the difference was not significant (*t*_9_ = 2.07, *P* = 0.069; Fig. 3). Adult pink salmon from the control group (*n* = 18; 10 females and 8 males), which were never exposed to PEA as juveniles, did not spend more time in either arm (*t*_17_ = 0.18, *P* = 0.86; Fig. 2a), did not cross through the inflow more frequently in either arm (*V* = 83, *n* = 18, *P* = 0.78; Fig. 2b), and did not spend a proportion of time in the PEA arm that differed from 0.5 (*t*_17_ = −0.22, *P* = 0.83; Fig. 3).

## Discussion

Our findings indicated that pink salmon alevins are able to imprint on the chemical composition of rearing water, prior to full absorption of the yolk sac and emergence from the gravel. While these results might be expected based on their life history, these are the first results to provide direct evidence that pink salmon imprint to chemical cues as alevins. Similar results might also be expected in other species of Pacific salmon, particularly chum salmon, which also do not rear in fresh water prior to their downstream migration^16^,^35^. Further studies on pink salmon that make use of a larger sample size could also lend evidence to better assess the accuracy of our findings.

There is substantial evidence that imprinting occurs during the PST in *Oncorhynchus* species^3^,^4^,^5^,^6^,^7^,^8^. The PST is characterized by endocrine, physiological, and behavioural changes that prepare salmon for life in the ocean^36^, including increases in gill Na+, K+ ATPase (ATPase) activity and circulating thyroxine levels linked to imprinting^12^,^13^. For many species with extensive freshwater rearing, the PST occurs months or years after emergence from the natal gravel, during a salmon’s final exposure to odours of its home river prior to seaward migration. For pink and chum salmon the transition to a saltwater environment often occurs as juveniles are leaving their natal site. Previous studies have indicated that just before emergence, pink salmon experience increases in ATPase and thyroxine levels reminiscent of the PST in other species^22^,^37^. In pink salmon this developmental period may be analogous to the PST and therefore may represent a similar sensitive period for imprinting.

Other Pacific salmon species with more extensive freshwater rearing can return to natal sites they only experienced as embryos, which suggests that imprinting may also occur prior to emergence in species other than pink and chum salmon. For example, sockeye salmon migrate to a nearby rearing lake immediately after emergence, indicating that imprinting during either the embryo or alevin stage is necessary for returning adults to navigate towards their incubation sites using olfactory directional cues. Indeed, sockeye salmon can demonstrate very fine-scale homing to a natal site only experienced as embryos^20^. If imprinting does not occur until the PST, returning adults could use olfactory directional cues to locate their rearing lake, but would presumably require some other type of directional cues to find their natal stream. Similarly, coho and Chinook (*O. tshawytscha*) salmon often make extensive migrations from their natal site prior to the PST, suggesting imprinting may occur before this period^38^,^39^.

Hasler and Scholz^3^ hypothesized that salmon imprint on a ‘bouquet’ of odours in their rearing water, and if true, the individual imprinting chemicals typically used in these studies (e.g. PEA, morpholine) would be just one component added to a more complex bouquet of odours. In many previous artificial imprinting experiments, adults were tested in water that differed from their rearing water such that the imprinted odorant was the only familiar cue available (e.g. refs 2 and 6). It is interesting to note that in our study, salmon were maintained in the same water source throughout their lives including the period of exposure to the imprinting odorant, rearing, and behavioural testing of adults. If the chemical composition of this water remained consistent through the duration of the study, the results of our experiment would indicate that the salmon distinguished the imprinted odour bouquet containing the artificial chemical PEA from the bouquet lacking PEA.

Previous studies in which a single imprinting odorant was added to a complex natural water in an attempt to increase homing fidelity to a hatchery produced mixed results^40^,^41^,^42^. In a series of hatchery experiments, salmon were either exposed to normal hatchery water or hatchery water containing the imprinted odorant morpholine prior to release. In one study, morpholine-exposed coho salmon returned to the hatchery in higher numbers than control fish when morpholine was added to the return ladder, suggesting that salmon had imprinted to and distinguished water with morpholine added^41^. However, in similar studies with coho and Chinook salmon, prior exposure to morpholine-scented hatchery water did not influence return rates when morpholine was added to the return ladder^40^,^42^.

Taking a different approach, Yamamoto and Ueda^43^ studied imprinting and homing responses of Osaru River chum salmon by identifying the amino acid profile of their natal river and testing the behavioural responses of Osaru River chum salmon to an artificial Osaru River water created by dissolving the same concentration and composition of amino acids to blank fresh water. They also created a second artificial river water that was identical except the most abundant amino acid was not added. Behavioural response tests showed the adult chum salmon preferred both artificial waters to waters from other rivers, and they did not distinguish the two replicates from one another. In other words, the removal of a single amino acid did not affect the response of the salmon to the chemical bouquet.

It is possible that the chemical composition of the water used for our experiment changed between the rearing period and the behavioural trials two years later, aside from PEA, which remained stable. Yamamoto *et al.*^44^ found that the concentrations of many amino acids in a salmon-bearing river in Hokkaido, Japan changes seasonally and annually. Of course, returning salmon are presumably able to recognize the overall odour of their natal water even if the specific chemical composition changes, and the intricacies of the specific chemicals involved in olfactory imprinting are simply not yet understood. Taken together, the previous research and our study suggest Pacific salmon may be able to detect and distinguish changes to even a single component of the olfactory bouquet, but their sensitivity to such changes, and how changes in chemical composition might occur in the natural environment, remain unknown.

Our finding with pink salmon provides evidence for the long-standing assumption that some Pacific salmon imprint on their natal water prior to the PST. It is possible that imprinting might occur prior to emergence in other species as well. It is also possible that the necessity for pink salmon to imprint earlier than their congenerics might contribute to reduced home stream fidelity, as the olfactory and central nervous systems may be relatively less developed at the time of imprinting. The timing of imprinting in different salmon species is not only important biologically but may have important implications for salmon hatchery rearing and release practices as well^17^. Further studies on the imprinting process in species such as chum salmon, which also must imprint at an early life stage before migrating to the ocean, and sockeye salmon, which may need to imprint prior to moving from their natal stream to their rearing lake, could determine whether species-level similarities or differences exist.

## Additional Information

**How to cite this article**: Bett, N. N. *et al.* Evidence of Olfactory Imprinting at an Early Life Stage in Pink Salmon (*Oncorhynchus gorbuscha*). *Sci. Rep.* **6**, 36393; doi: 10.1038/srep36393 (2016).

**Publisher’s note**: Springer Nature remains neutral with regard to jurisdictional claims in published maps and institutional affiliations.
